# Short‐term impact of fire on the total soil microbial and nitrifier communities in a wet savanna

**DOI:** 10.1002/ece3.7661

**Published:** 2021-07-01

**Authors:** Tharaniya Srikanthasamy, Sébastien Barot, Fulgence K. Koffi, Kevin Tambosco, Yoan Marcangeli, David Carmignac, Aya Brigitte N'Dri, Jonathan Gervaix, Xavier Le Roux, Jean‐Christophe Lata

**Affiliations:** ^1^ Sorbonne Université, Université de Paris UPEC CNRS INRAE IRD UMR 7618 Institute of Ecology and Environmental Sciences – Paris, iEES Paris Paris France; ^2^ IRD Sorbonne Université CNRS INRAE Université de Paris UPEC UMR 7618 Institute of Ecology and Environmental Sciences – Paris, iEES‐Paris Paris France; ^3^ UFR‐SN / Research Station of Lamto (CRE) Research Pole Environment and Sustainable Development, Nangui Abrogoua University (ex University of Abobo‐Adjamé), Abidjan, Côte d'Ivoire Abidjan Ivory Coast; ^4^ INRA CNRS Université de Lyon Université Lyon 1 Laboratoire d'Ecologie Microbienne UMR INRA 1418 UMR CNRS 5557 Villeurbanne France; ^5^ Department of Geoecology and Geochemistry Institute of Natural Resources Tomsk Polytechnic University Tomsk Russia

**Keywords:** AOA, AOB, Biological Nitrification Inhibition (BNI), burning, nitrification, perennial grasses, savanna, trees

## Abstract

Savannas are characterized by the coexistence of grasses and trees. Fires are critical for their coexistence, because they decrease the survival of tree seedlings and saplings and their recruitment to the adult stage. In some humid savannas, perennial grasses inhibit nitrification and trees stimulate nitrification, which likely favors coexistence between trees and grasses. However, fires may influence plant capacity to control nitrogen cycling, which could subsequently influence tree–grass coexistence and savanna nitrogen budget. Therefore, we sampled soil in a humid savanna of Ivory Coast under the dominant nitrification‐inhibiting grass species and the dominant nitrification‐stimulating tree species and under bare soil before and after (i.e., 5 days) fire during the long dry season. We quantified the total microbial and nitrifier abundances and transcriptional activities and the nitrification enzyme activity. Fire decreased soil water content, probably by increasing evaporation and, maybe, by triggering the growth of grasses, and increased soil ammonium availability likely due to ash deposition and increased mineralization. Fire did not impact the total archaeal, bacterial, or fungal abundances, or that of the nitrifiers. Fire did not impact archaeal transcriptional activity and increased bacterial and fungal total transcriptional activities. In contrast, fire decreased the archaeal nitrifier transcriptional activities and the nitrification enzymatic activity, likely due to the often reported resumption of the growth of nitrification‐inhibiting grasses quickly after the fire (and the subsequent increase in root exudation). These results pave the way for a better understanding of the short‐term effects of fire on nitrogen cycling and tree–grass competition for nitrogen.

## Graphical abstract

In some humid savannas, perennial grasses inhibit nitrification and trees stimulate nitrification, which likely favors coexistence between trees and grasses. Fires may influence plant capacity to control nitrogen cycling. In this study, after the fire, the archaeal nitrifier transcriptional activities and the nitrification enzymatic activity decreased, likely due to the often reported resumption of the growth of nitrification‐inhibiting grasses quickly after the fire (and the subsequent increase in root exudation).

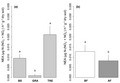

## 1 INTRODUCTION

Savannas are characterized by the coexistence of grasses and trees (Sankaran et al., 2004), and they cover 12%–13% of global terrestrial areas (Rutten et al., 2016). Tree/grass coexistence is usually explained by disturbances or resource partitioning (Barot & Gignoux, 2004; House et al., 2003; Sankaran et al., 2004). Herbivory and fires favor tree–grass coexistence (Sankaran et al., 2004), because they decrease the survival of tree seedling and sapling and their recruitment to the adult stage (Menaut & Gignoux, 1990). In humid savanna, grasses are mostly perennial tussock grasses that are well adapted to fires: They store photosynthates in their roots at the end of the rainy season (Abbadie & Lata, 2006; Aerts, 1996; Ratnam et al., 2008). The aboveground biomass of these tussocks burns during the dry season and they quickly resume their postfire growth at the beginning of the next rainy season. Fires are particularly influential for the maintenance of West African savannas (Rose Innes, 1972) as they impact their whole functioning, including carbon (C) and nitrogen (N) cycles and soil properties. Different factors, such as fire intensity, climate conditions, topography, and landscape heterogeneity, change the impact of fires on soil characteristics and functioning (Francos et al., 2018; Wright & Clarke, 2008). For example, season and soil moisture have direct impacts on temperature profiles during a fire. In dry seasons, soil heating remains elevated for longer periods while when soil moisture is high, it greatly reduces soil heating (Wright & Clarke, 2008).

It is possible to distinguish long‐term impacts of fires (comparing plots with and without fires for several years, e.g., Riddell et al., 2012) from short‐term impacts (comparing plots just before and after a fire, Gómez‐Rey & Gonzalez‐Prieto, 2013). In the Mediterranean or arid and semi‐arid temperate zones, different short‐ and long‐term impacts of fires have been documented. On the short‐term, fires modify soil pH that tends to increase immediately after fire (Muñoz‐Rojas et al., 2016; Rodríguez et al., 2017; Yinghua et al., 2012). This is due to the release of alkaline ions from K and Na oxides, hydroxides, and carbonates in the ashes (Xue et al., 2014) and to the denaturation of organic acids by heating (Scharenbroch et al., 2012). The mineral nutrient availability also increases (Muñoz‐Rojas et al., 2016) due to ashes that quickly release their mineral nutrients after fires and to an increased mineralization of soil organic matter (Xue et al., 2014). These fire impacts on mineral nutrient and C cycling can also alter both soil microbial activities and functional diversity (Yang et al., 2020; Yinghua et al., 2012). This is particularly true in the top 15 cm of soil where most aerobic microbial activities occur (Docherty et al., 2012) and where microbial communities can be impacted by severe fires (Bárcenas‐Moreno et al., 2011).

At least, three mechanisms likely impact soil microorganisms during and immediately after fires: (a) Temperature increases in the first centimeters of soil (Certini, 2005; Gillon, 1983; Neary et al., 1999). For instance, Yinghua et al. (2012) reported that, in the arid and semi‐arid temperate zone, soil microbial biomass decreases immediately after fire because of the heat‐induced microbial mortality. Similarly, Goberna et al. (2012) reported that archaeal community structure changes immediately following prescribed fire in Mediterranean shrublands, but to a lesser extent than bacteria and fungi and without being linked to changes in ammonium availability or soil pH. (b) Fires lead to losses of organic matter (litter at the soil surface is burnt as well as some soil organic matter in the first centimeters) and of mineral N by volatilization. In the Mediterranean area, Guerrero et al. (2005) reported that fungi seem to be more sensitive to fire than bacteria, likely due to the reduction of the availability of C, N, and some organic compounds such as cellulose and to the fact that bacteria tend to be more resistant to the direct effect of fire and heat than fungi (Guerrero et al., 2005; Hart et al., 2005; Rodríguez et al., 2017). In contrast, the fire effects on soil archaeal communities are poorly documented (Goberna et al., 2012; Mikita‐barbato et al., 2015). (c) In frequently burnt savannas, perennial grasses tend to resume their growth just after fires (Gignoux et al., 2006), which suggests that fires trigger root growth and root exudation. This could result in differences in microbial communities without marked changes in soil physicochemical properties.

Fire might also impact specifically soil N‐cycling communities. Heating during fire can volatilize N in the topsoil, which decreases N availability and could favor archaeal (AOA) over bacterial (AOB) nitrifiers since low and high N availabilities respectively favor AOA and AOB in savanna soils (Assemien et al., 2017). The AOA could also be favored by higher archaeal resistance to high temperatures (Goberna et al., 2012). The concentration of microbial enzymes involved in organic N decomposition can increase or decrease after fires (Docherty et al., 2012). The same authors also reported that fire had no significant effect on the abundance of bacterial nitrifier communities in a Mediterranean grassland of California. However, Niboyet et al. (2011) showed that fire decreased nitrification and Yang et al. (2020) reported decreased abundances of most functional genes associated with N cycling after fire for the same site. So far, the short‐term impacts of fires on soil microorganisms have never been studied in savannas, especially in West Africa.

A specificity of the dominant perennial grass species in West African savannas is that they inhibit nitrification through their Biological Nitrification Inhibition (BNI) activities (Lata et al.,^1999^, ^2000^, ^2004^; Srikanthasamy et al., 2018; Subbarao et al., 2009). Grass roots exudate into the soil nitrification‐inhibiting molecules that can block the bacterial ammonia oxidation pathway (Srikanthasamy et al., 2018; Subbarao et al., 2009) and probably the archaeal ammonia oxidation pathway (Sarr et al., 2020; Srikanthasamy et al., 2018). Besides, in humid savanna such as Lamto (Ivory Coast), trees probably stimulate nitrification through a mechanism that has so far not been identified (Srikanthasamy et al., 2018). As a result, grasses and trees create heterogeneity in N cycling, which could facilitate their coexistence (Konaré et al., 2019). In this type of savanna, annual fires could thus impact nitrification and nitrifying microbial communities through direct effects on soils and indirect effects on grass and tree growth, i.e. altering root growth and exudation. In the case of a root‐mediated short‐term effect of fire, one can expect a stimulation of most soil microbial communities through enhanced exudation, but an increased inhibition in the rhizosphere of BNI‐grass species. Because trees and grasses have opposite effects on nitrification, we can thus predict significant interaction effect between the vegetation cover and the short‐term effect of fire. In any case, while the impact of trees and grasses on microbial communities and nitrification has already been described in the Lamto savanna (Srikanthasamy et al., 2018), the short‐term effect of the annual fire on soil microbial functioning has hardly been studied in any savanna (but see Andersson et al., 2004). Our study therefore aims at answering the following questions: What is the short‐term impact of fire on (a) soil physicochemical characteristics, (b) the total abundances of archaeal, bacterial and fungal communities, (c) nitrifier communities, their activities, and the subsequent nitrification rate? (d) Does fire modify the influence of trees and grasses on nitrifier communities and nitrification?

## 2 MATERIALS AND METHODS

### 2.1 Study site

The Lamto reserve is located in Ivory Coast, West Africa (6°9’–6°18′ N; 5°15′–4°57′ W). The vegetation is a mosaic of savannas with various tree densities and gallery forests, developed on tropical ferrugineous soils with a superficial gravelly horizon. These soils are sandy (ca. sand 77%; silt 14%; clay 9%) and with a bulk density of ca. 1.65 (Abbadie & Menaut, 2006). Temperatures are relatively constant throughout the year (27°C on average). Four seasons can be distinguished: (a) a long dry season from December to February; (b) a long wet season from March to July; (c) a short dry season in August; and (d) a short wet season from September to November. Annual precipitation in 2017 was 1100.5 mm, close to the long‐term average, i.e. 1192, calculated from 1962 to 1995 (data from the Geophysical Station of Lamto). Prescribed fire burns the savanna annually, because fire is seen as a tool to manage the equilibrium between grasses and trees in humid savannas. The fire is set to Lamto savanna every year around January 15. Typically, the fire spread rate is about 0.125 m/s and its intensity is about 3500 kW m^−1^ (N'Dri et al., 2018), and the fire burns all the aboveground grass biomass. Temperature at soil surface does not exceed 60°C during fires and decreases rapidly with soil depth (Gillon, 1983).

### 2.2 Soil sampling

Tree and grass individuals are spatially well separated. Perennial grass individuals form large tussocks separated by at least 20 cm of bare soil. Trees grow separately or in clumps but clumps were avoided for the sampling. We therefore focused on rhizospheric soil directly sampled in the rhizosphere of identified grass and tree individuals. Soil was sampled during the dry season (January 2017, 107.7 mm of precipitation in the previous 2 months and 0 mm in the last 15 days) 5 days before (BF) and 5 days after (AF) fire in an open shrub savanna. Samples were taken under the tussocks of five individuals of the dominant BNI grass *Hyparrhenia diplandra* (HD), under the canopy of five individuals of the dominant tree species *Crossopteryx febrifuga* (CF) and in bare soil patches (BS—five replicated 2 × 2 m patches of soil without vegetation that had been weeded for 1 year before the sampling). Soil sampling below tree canopy was achieved between grass tussocks (that are less abundant under tree canopy). Grass tussocks were chosen to have similar basal diameter (ca. 18 cm), and trees were selected to have similar diameter at breast height (ca. 20 cm). The choice of plant individuals was made randomly on a surface of *ca*. 10 ha, and local sources of heterogeneity (termite mounds, small depressions, rocks) were avoided. The minimum distance between two soil samples was 10 m (confirmed by GPS coordinates at each sampling site).

Soil moisture was measured in the field before sampling with a ThetaProbe ML3 (Delta‐T Devices) previously calibrated on oven‐dried soils. For each of the 15 samples (5 samples for the grass species, tree species, and bare soil), soil (about 1 kg) was collected from the 0–15 cm layer with an auger (10 cm in diameter). Around 10 g of each sample was placed in cryotubes and directly fixed in liquid N in the field for RNA‐based analyses. The rest of each soil sample was stored at 4°C for a very short period during transport to the field station. These samples were subsequently sieved (2 mm) and homogenized, and 200 g of soil was stored at −20°C for molecular biology analyses and the measurements of nitrifying enzyme activities. The remaining soil was immediately air‐dried in the shade and stored at ambient temperature for physicochemical analyses. Roots were collected from all soil samples through dry sieving. Fine root densities varied between 2.06 and 9.08 g dry root dm^−3^ dry soil under grasses, between 0.89 and 2.06 g dry root dm^−3^ dry soil under trees, and between 0 and 0.30 g dry root dm^−3^ dry soil under bare soil. The aboveground biomass of each grass tussock was collected and dried at 50°C before weighing and was around 1.5 g dry matter tussock^−1^. Soil moisture was measured in the field before sampling with a ThetaProbe ML3 (Delta‐T Devices) previously calibrated on oven‐dried soils.

### 2.3 Microbial abundances and transcriptional activities

#### 2.3.1 Total nucleic acids extraction

Total ribonucleic acids were extracted from 4 g of soil (wet mass) with the RNA PowerSoil® Total RNA Isolation Kit from MO BIO Laboratories, in combination with the FastPrep FP120 bead beating system (Bio‐101, Inc., Ca, USA) according to the manufacturer's instructions. DNase treatment was performed using the Ambion® TURBO DNA‐free™ DNase Treatment and Removal Reagents protocol, and the absence of contaminant DNA was checked on agarose gel. RNA was quantified using the Qubit™ RNA HS Assay Kit. All RNA samples were normalized to a similar concentration (about 8 ng/µl) before the reverse transcription step. The reverse transcription was done using SuperScript® III First‐Strand Synthesis System for RT‐PCR from Invitrogen. Before further analyses, all extracted RNA samples were stored at −80°C and cDNA samples were stored at −20°C.

Total deoxynucleic acids were extracted from 0.5 g of soil (wet mass) with a Bio‐101 FastDNA Spin kit in combination with the FastPrep FP120 bead beating system (Bio‐101, Inc., Ca, USA) according to the manufacturer's instructions. Bulk total DNA was purified by elution through Geneclean Turbo columns (MP Biomedicals, CA, USA) according to the manufacturer's instructions. DNA was quantified using the Qubit™ dsDNA HS Assay Kit. All extracted DNA samples were stored at −20°C before being analyzed.

#### 2.3.2 Real‐time PCR quantification

The abundances of total bacteria (16S rRNA), total archaea (16S rRNA), total fungi (18S rRNA), nitrifying archaea (*amoA‐AOA*), and nitrifying bacteria (*amoA‐AOB*) were determined by Real‐Time PCR (CFX96 Real‐Time System, Bio‐Rad, France) with specific primer sets (Table S1). Quantification was made with the SYBR Green dye. The real‐time PCR assay was carried out in a 20 µl reaction volume containing SoAdvanced SYBRGreen Supermix (2X, Bio‐Rad) and 1.25 µl of bovine albumin serum (2 mg ml^‐1^). Two independent quantitative PCR assays were performed for each gene to check repeatability, and the mean of the two obtained values was used in statistical analyses. Standard curves were obtained using serial dilutions of linearized plasmids containing the studied genes. Average PCR efficiency was of 91.7%, 95.2%, 85.2%, 84.1%, and 91.8% for archaeal 16S rRNA, bacterial 16S rRNA, fungal 18S rRNA, *amoA‐*AOA, and *amoA*‐AOB genes, respectively. Melting curves were analyzed using the Dissociation Curve Analysis Software (Applied Biosystems). Results were expressed as gene copy numbers per gram of dry soil. All ratios were calculated in the same way as the following example: Bacteria:Fungi ratio = copy number of bacterial 16S rRNA/copy number of fungal 18S rRNA.

#### 2.3.3 Nitrifying enzyme activity assays

Potential nitrification rates were measured through nitrifying enzyme activity (NEA) assays that correspond to short‐term laboratory incubations under nonlimiting conditions. This approach dampens short‐term variations induced in the field by climate or other environmental factors and allows detecting very low rates of nitrification (Attard et al., 2011; Lata et al., 1999). Frozen soil samples were placed at ambient room temperature for 2 hr before the analyses. Frozen samples were preferred to fresh or dried samples because (a) the time between sampling and analysis (including transportation) was too long to use fresh soil, (b) previous studies showed that for soil enzyme activity studies, freezing was a preferable storage method (Wallenius et al., 2010), and (c) preliminary tests on fresh versus frozen samples on our soils showed no discrepancy (JC Lata and F Poly, unpublished).

Soil NEA was measured according to Dassonville et al. (2011) as the linear rate of production of nitrate during a 72‐hr incubation. Subsamples (3 g equivalent dry soil) were incubated with 6 ml of a solution of (NH_4_)_2_SO_4_ (22 μg N‐NH_4_^+^ g^−1^ dry soil). Distilled water was added in each sample to reach 30 ml of total liquid volume in flasks. Soil nitrate content was measured after 5, 24, 48, and 72 hr of incubation under constant agitation (140 rpm) by ion chromatography (ICS‐900, Dionex, Salt Lake City, USA).

### 2.4 Soil physicochemical characteristics

Soil pH was measured in water (5:1 v/v water:soil) with a pH meter (Thermo Scientific Orion™ Star A211, and Orion™ PerpHecT™ ROSS™ Combination Micro Electrode) according to the NF ISO 10390 standard. Mineral N was extracted from 2 g frozen soil by adding 2 M KCl solution (soil:solution = 1:4). Nitrate was reduced to nitrite and nitrite, and ammonium concentrations (expressed as mg N‐NO_3_^‐^ and N‐NH_4_^+^ g^−1^ dry soil) were then measured with a continuous‐flow N analyzer (SKALAR, San Plus System, Breda, the Netherlands). Total C and N contents in soils (expressed as %) were measured using a CHN Elemental Analyzer (FlashEA 1112 Series, Thermo Electron Corporation, Netherlands) after grinding at 80 µm.

### 2.5 Statistical analyses

All statistical analyses were performed using R software (R Core Team 2016). The normality and homoscedasticity of the residuals of all linear models were tested with Shapiro–Wilk and Bartlett tests, respectively. Data were log‐transformed, or square root‐transformed or boxcox‐transformed in case of significant deviation from normality and homoscedasticity. All measured variables were analyzed using an ANOVA testing for the effect of the vegetation cover, the fire (sampling before or after the fire), and the interaction between the two. In case of a significant effect of the interaction between vegetation cover and fire, all combinations of the two treatments were compared using post hoc tests (Tukey honestly significant difference test). When the interaction was not significant, only the modalities of the significant simple effects were compared with post hoc tests (Tukey honestly significant difference test). We tested the relation between NEA and the abundances of nitrifier communities (DNA and transcripts) using a linear regression. For all tests, the null hypothesis was rejected for *p* < 0.05 and significance is represented as follows: *** when *p* < 0.001; ** for 0.001 < *p* < 0.01; * when 0.01 < *p* < 0.05.

Data were finally analyzed using principal component analysis (PCA) using the ADE‐4 package (Thioulouse et al., 1997). Monte Carlo permutation test was also performed to examine the relationship between the nine geochemical properties and bacterial community composition in this Arctic lake area. Monte Carlo permutation test was also performed to examine the relationship between the physicochemical properties and the covers.

## 3 RESULTS

### 3.1 Nitrifying enzyme activity

**FIGURE 1:**
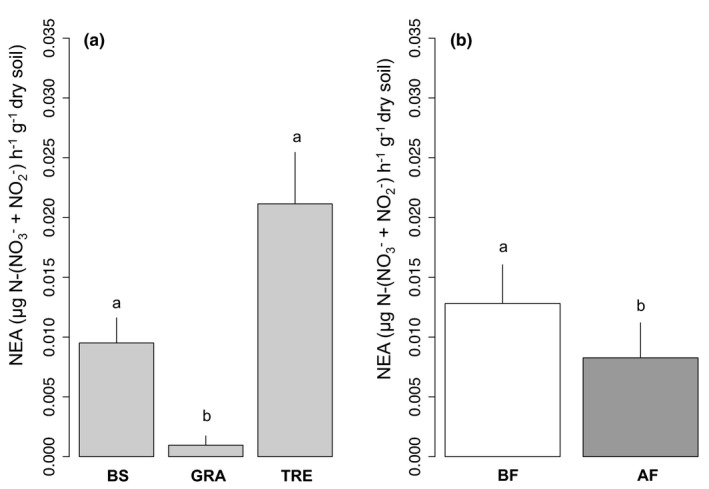
Soil nitrifying enzyme activities (NEA) according to the vegetation cover (a) and the fire treatment (b): bare soil (BS), dominant grass species *Hyparrhenia diplandra* (GRA) and dominant tree species *Crossopteryx febrifuga* (TRE), before fire (BF, in white) and after fire (AF, in gray), light gray = mean effect of the vegetation cover. Means and *SE*s were calculated from ten replicates, and small letters indicate statistical difference (*p* < 0.05)

The nitrifying enzyme activity (NEA) was affected by the vegetation cover and the fire treatment (Figure 1; Table 1; see also all mean values in Table S2). Soil NEA was higher under tree (2.11 ± 0.43 × 10^–2^ µg N‐(NO_3_^‐^ + NO_2_^‐^) h^−1^ g^−1^ dry soil; *p* < 0.001) and bare soil (9.51 ± 2.10 × 10^–3^ µg N‐(NO_3_^‐^ + NO_2_^‐^) h^−1^ g^−1^ dry soil; *p* < 0.01) than under grass (0.96 ± 0.78 × 10^–3^ µg N‐(NO_3_^‐^ + NO_2_^‐^) h^−1^ g^−1^ dry soil). Soil NEA was overall higher before fire (1.28 ± 0.40 × 10^–2^ µg N‐(NO_3_^‐^ + NO_2_^‐^) h^−1^ g^−1^ dry soil) than after (0.83 ± 0.36 × 10^–2^ µg N‐(NO_3_^‐^ + NO_2_^‐^) h^−1^ g^−1^ dry soil). No interaction between vegetation cover and fire was observed, due to the variability of the response of the 3 types of vegetation cover: NEA decreases after fire for bare soil (from 14.62 (before fire) to 4.40 (after fire) µg N‐(NO_3_^‐^ + NO_2_^‐^) h^−1^ g^−1^ dry soil), becomes virtually zero under grass and remains stable in tree (21.89 (before fire) vs. 20.40 (after fire) µg N‐(NO_3_^‐^ + NO_2_^‐^) h^−1^ g^−1^ dry soil).

**TABLE 1:** Statistical results (ANOVA) of fire effect and vegetation cover on different soil physicochemical characteristics (NEA = nitrifying enzyme activity), on the abundance and transcripts of total microbial communities and nitrifying communities (archaeal nitrifier (AOA) and bacterial nitrifier (AOB))

	Variable	Transformation	Cover (*df* = 2)		Fire (*df* = 1)		Cover * fire (*df* = 2)	
			*F*‐value	*p*‐value	*F*‐value	*p*‐value	*F*‐value	*p*‐value
Soil properties	NEA	Square root	18.236	<0.001	4.229	<0.05	0.916	0.414
	Nitrate (N‐NO_3_^‐^)		0.260	0.773	0.0001	0.992	4.189	<0.05
	Ammonium (N‐NH_4_^+^)		9.661	<0.001	4.475	<0.05	2.519	0.101
	Soil Water Content (SWC)		2.359	0.116	30.430	<0.001	5.160	<0.05
	pH		8.198	<0.01	0.072	0.782	0.013	0.987
	Total nitrogen		6.260	<0.01	0.291	0.591	0.780	0.470
	Total carbon		4.369	<0.05	0.036	0.851	1.085	0.354
Total microbial abundance and activity	Total archaea (16S rRNA)	log	0.209	0.812	0.637	0.432	0.778	0.471
	Total archaeal transcripts (16S rRNA)		2.890	0.074	0.260	0.614	2.719	0.086
	Total bacteria (16S rRNA)	Square root	0.535	0.592	0.008	0.929	0.767	0.476
	Total bacterial transcripts (16S rRNA)	Square root	4.810	<0.05	30.688	<0.001	3.778	<0.05
	Total fungi (18S rRNA)		9.383	<0.001	51.514	0.476	0.236	0.792
	Total fungal transcripts (18S rRNA)	Square root	9.383	<0.01	51.514	<0.001	4.992	<0.01
	Bacteria:Fungi ratio	log	33.043	<0.001	0.316	0.579	0.589	0.563
	Bacterial:Fungal transcripts ratio	Boxcox transformation *λ* = −0.465	8.722	<0.01	72.190	<0.001	0.634	0.539
Nitrifier abundance and activity	AOA		10.826	<0.001	0.288	0.596	4.190	0.059
	AOA transcripts	Square root	1.890	0.170	16.418	<0.001	3.841	0.068
	AOB	Square root	5.794	<0.01	1.799	0.083	1.920	0.170
	AOA:(AOA + AOB) ratio		3.933	0.066	2.350	0.145	2.768	0.117
	AOA:total Archaea ratio		3.933	0.066	1.603	0.236	1.934	0.185
	AOA:Archaeal transcripts ratio		1.270	0.309	0.618	0.445	1.160	0.298
	AOB:Bacterial ratio	log	6.619	<0.01	1.396	0.249	1.032	0.373

Note

The interaction between fire and vegetation cover was removed when not significant.

### 3.2 Total microbial abundances and transcriptional activities

The total archaeal abundance and transcripts were not affected by the vegetation cover and the fire treatment (Table 1; see also all mean values in Table S3 for total abundances and Table S4 for transcripts).

**FIGURE 2:**
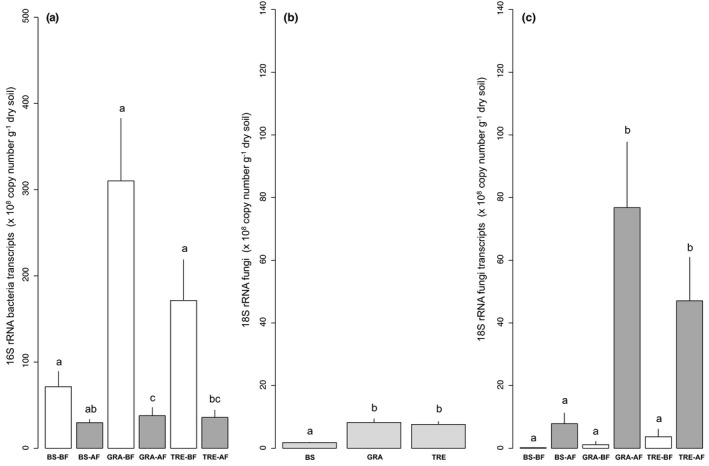
Transcripts of total bacteria (a) and abundances and transcripts of total fungi (b, c) according to the vegetation cover and the fire treatment: bare soil (BS), dominant grass species *Hyparrhenia diplandra* (GRA) and dominant tree species *Crossopteryx febrifuga* (TRE), before fire (BF, in white) and after fire (AF, in gray), light gray = mean effect of the vegetation cover. Means and *SE*s were calculated from five replicates for the interaction effect and ten replicates for the vegetation cover effect, and small letters indicate statistical difference (*p* < 0.05)

The total bacterial abundance was not affected by the vegetation cover and the fire treatment (Table 1; see also all mean values in Table S3 for total abundances and Table S4 for transcripts). The total bacterial transcripts were affected by the vegetation cover, the fire treatment, and the interaction between the two (Figure 2a and Table 1). They were higher for grass‐after fire (3.10 ± 0.72 × 10^10^ copies g^−1^ dry soil) than for grass‐before fire (3.78 ± 0.85 × 10^9^ copies g^−1^ dry soil; *p* < 0.001) and higher for tree‐after fire (1.71 ± 0.47 × 10^10^ copies g^−1^ dry soil) than for tree‐before fire (3.58 ± 0.85 × 10^9^ copies g^−1^ dry soil; *p* < 0.05). They were also higher for grass‐after fire than for bare soil‐after fire (7.14 ± 2.44 × 10^9^; *p* < 0.01).

The total fungal abundance was affected by the vegetation cover (Figure 2c and Table 1; see also all mean values in Table S3 for total abundances and Table S4 for transcripts). It was higher under grass (8.22 ± 1.18 × 10^8^ copies g^−1^ dry soil; *p* < 0.001) and tree (7.62 ± 0.90 × 10^8^ copies g^−1^ dry soil; *p* < 0.001) than under bare soil (1.81 ± 0.12 × 10^8^ copies g^−1^ dry soil). The total fungal transcripts were affected by the vegetation cover, the fire treatment, and the interaction between the two (Figure 2c and Table 1). They were higher for grass‐after fire (7.68 ± 2.09 × 10^9^ copies g^−1^ dry soil) than for grass‐before fire (0.925 ± 0.83 × 10^8^ copies g^−1^ dry soil; *p* < 0.001) and higher for tree‐after fire (4.71 ± 1.42 × 10^9^ copies g^−1^ dry soil) than for tree‐before fire (3.68 ± 2.40 × 10^8^ copies g^−1^ dry soil; *p* < 0.01). Finally, the total fungal transcripts were higher for grass‐after fire (*p* < 0.001) and tree‐after fire (*p* < 0.05) than for bare soil‐after fire.

The bacteria:fungi ratio was affected by the vegetation cover. The ratio was higher under bare soil (9.91 ± 0.58) than grass (2.28 ± 0.36; *p* < 0.001) and tree (2.80 ± 0.40; *p* < 0.001). The bacterial:fungal transcripts ratio was affected by the vegetation cover and the fire. The bacterial:fungal transcripts ratio was higher under bare soil (1.14 ± 0.78 × 10^3^) than grass (7.82 ± 4.41 × 10^1^) and tree (1.06 ± 0.81 × 10^2^). The ratio was higher before (9.31 ± 5.56 × 10^2^) than after the fire (6.44 ± 1.40; *p* < 0.001).

### 3.3 Nitrifier abundances and transcriptional activities

**FIGURE 3:**
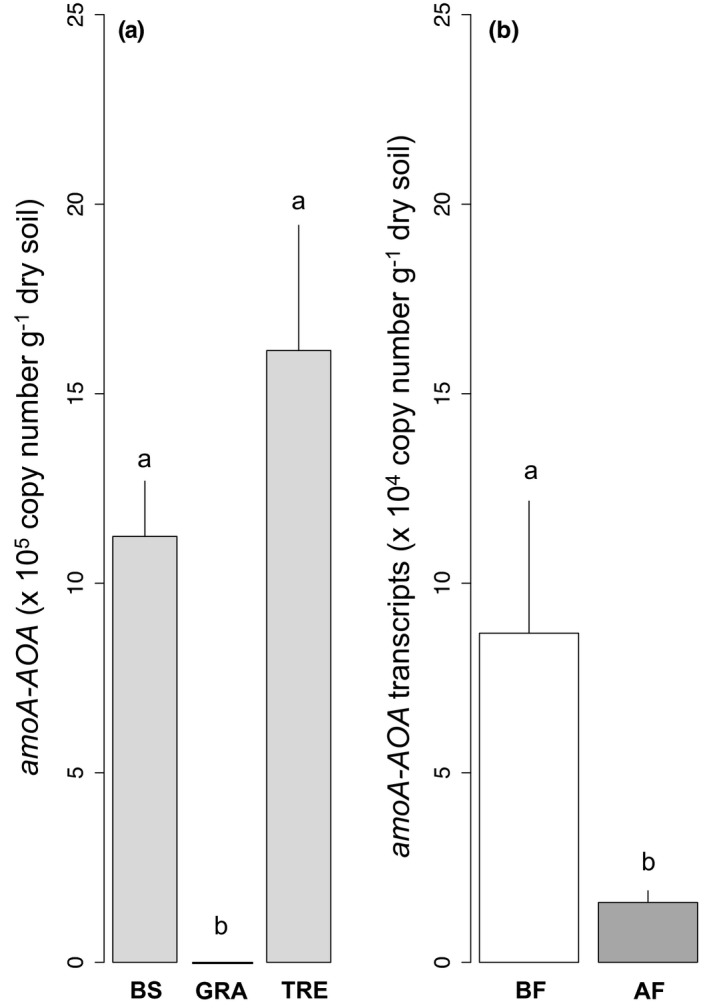
Abundances (a) and transcripts (b) of archaeal‐AOA amoA genes according to the vegetation cover and the fire treatment: bare soil (BS), dominant grass species *Hyparrhenia diplandra* (GRA) and dominant tree species *Crossopteryx febrifuga* (TRE), before fire (BF, in white) and after fire (AF, in gray), light gray = mean effect of the vegetation cover. Means and *SE*s were calculated from ten replicates for the vegetation cover effect and fifteen replicates for the fire effect, and small letters indicate statistical difference (*p* < 0.05)

The archaeal *amoA*‐AOA gene abundance was affected by the vegetation cover (Figure 3a and Table 1; see also all mean values in Table S3 for N genes abundances and Table S4 for transcripts). It was higher under bare soil (1.12 ± 0.21 × 10^6^ copies g^−1^ dry soil; *p* < 0.01) and tree (1.45 ± 0.34 × 10^6^ copies g^−1^ dry soil; *p* < 0.001) than under grass (00.00 ± 00.00 copies g^−1^ dry soil). The archaeal *amoA*‐AOA transcripts were impacted by the fire treatment and were higher before fire (8.10 ± 3.30 × 10^4^ copies g^−1^ dry soil) than after (7.37 ± 2.52 × 10^3^ copies g^−1^ dry soil; Figure 3b and Table 1).

**FIGURE 4:**
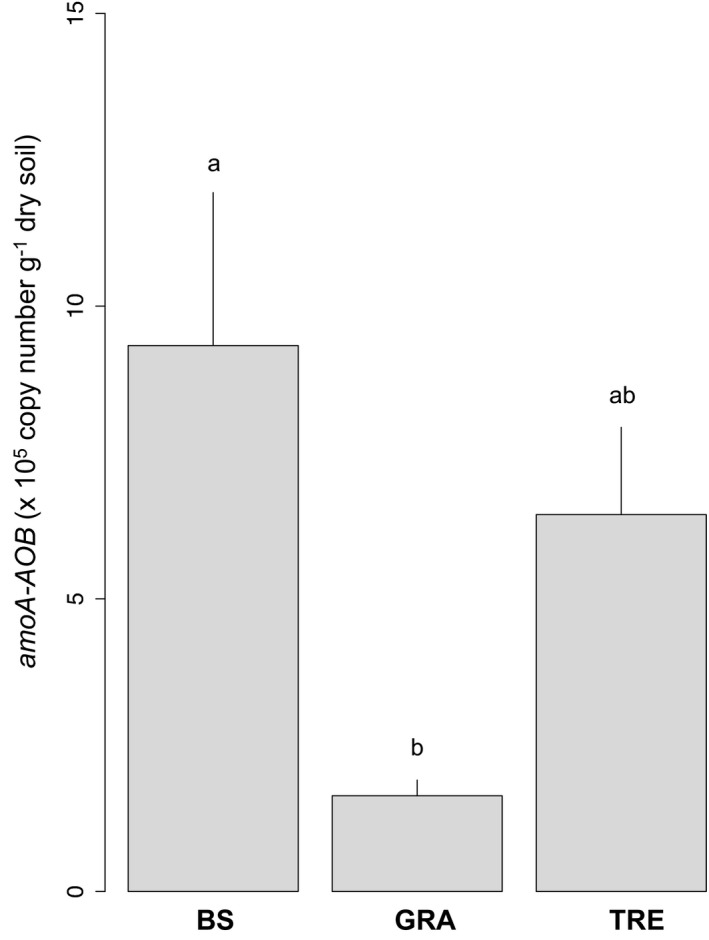
Abundances of bacterial‐AOB amoA genes according to the vegetation cover: bare soil (BS), dominant grass species *Hyparrhenia diplandra* (GRA) and dominant tree species *Crossopteryx febrifuga* (TRE). Means and *SE*s were calculated from ten replicates, and small letters indicate statistical difference between vegetation covers (*p* < 0.05)

The bacterial *amoA*‐AOB gene abundance was affected by the vegetation cover (Figure 4 and Table 1). It was higher under bare soil (9.33 ± 2.61 × 10^5^ copies g^−1^ dry soil; *p* < 0.01) than under grass (1.47 ± 0.29 × 10^5^ copies g^−1^ dry soil). The bacterial *amoA*‐AOB transcripts were under the detection limit for all samples but for a single sample, under a grass after the fire (2.4 × 10^4^ copies g^−1^ dry soil).

The AOB:total bacteria abundance ratio (Table 1) was only affected by the vegetation cover (*p* < 0.01). The values for tree (3.61 ± 0.77 × 10^–4^; *p* < 0.01) and bare soil (5.45 ± 1.55 × 10^–4^; *p* < 0.01) were higher than for grass (9.54 ± 0.96 × 10^–5^). No significant impact of the vegetation cover, the fire, or their interaction was observed for AOA:(AOA + AOB) ratio, AOA:total archaea abundance ratio, and AOA:Archaeal transcripts ratio. Finally, no significant relationship was observed between NEA and nitrifier abundance or the amount of transcripts.

### 3.4 Soil physical and chemical characteristics

The pH was only affected by the vegetation cover (*p* < 0.01; Table 1; see also all physicochemical mean values in Table S2). It was slightly higher under tree (tree, 6.88 ± 0.03) than under grass (grass, 6.65 ± 0.05; *p* < 0.05) and bare soil (bare soil, 6.69 ± 0.04; *p* < 0.05). The pH of grass and bare soil was not significantly different. The soil water content (SWC) was affected by the fire treatment (*p* < 0.001) and the interaction between vegetation cover and fire (*p* < 0.05). The soil water content was higher before (before fire; 14.80 ± 0.74%) than after (after fire; 9.55 ± 0.85%) the fire. In particular, the soil water content was higher for bare soil‐before fire (15.66 ± 1.10%) than for bare soil‐after fire (7.00 ± 0.63%; *p* < 0.001).

The N‐NH_4_^+^ content was affected by the vegetation cover (*p* < 0.001; Table 1) and the fire treatment (*p* < 0.05). It was higher under tree (7.10 ± 0.37 × 10^–3^ mg g^−1^ dry soil; *p* < 0.01) and grass (6.64 ± 0.36 × 10^–3^ mg g^−1^ dry soil; *p* < 0.05) than under bare soil (5.39 ± 0.19 × 10^–3^ mg g^−1^ dry soil). It was higher after fire (6.72 ± 0.38 × 10^–3^ mg g^−1^ dry soil) than before (6.03 ± 0.21 × 10^–3^ mg g^−1^ dry soil; *p* < 0.05). The N‐NO_3_^−^ content was not affected by the vegetation cover and the fire treatment. The total N content was affected by the vegetation cover (*p* < 0.01). It was higher under tree (0.075 ± 0.004%) than under bare soil (0.053 ± 0.005%; *p* < 0.01).

The total C content was affected by the vegetation cover (*p* < 0.05; Table 1). It was higher under tree (1.06 ± 0.06%) than under bare soil (0.80 ± 0.07%; *p* < 0.05). Overall, the only significant differences between soil properties before and after fire were for soil humidity and N‐NH_4_^+^.

The first axis of the PCA (Figure S1) denotes soil fertility, fertility increasing with positive values. The second axis of the PCA denotes the opposition between nitrate, soil water content, pH, and NEA on the one hand (negative part of the axis) and total carbon, total nitrogen, and the ammonium on the other hand (positive part of the axis). Nitrate is positively correlated with NEA, and these two variables are not correlated to ammonium and total nitrogen and carbon contents.

The different plant covers are significantly different (permutation test, *p* = 0.01; Figure S2). The comparison between plant covers opposes trees to grasses with tree being on the fertility side. Finally, the comparison between the situation before and after fire is also significant (permutation test, *p* = 0.01; Figure S3) and opposes the soil before fire with higher nitrification, nitrate, water content, and pH, to the soil after fire with lower values.

## 4 DISCUSSION

In our study, the soil water content decreased after the fire, which can be explained by two mechanisms: (a) The increase in soil temperature during fire, the decrease in soil protection by plant biomass after fire, and the decrease in surface albedo (Snyman, 2015) could increase evaporation. (b) Grasses resume their growth very quickly after fire (grass leaves already had a height of ca. 5 cm 5 days after fire, when soil was sampled, personal observation and Gignoux et al., 2006), which leads to an increase in evapotranspiration. Furthermore, pH slightly changed with the vegetation cover, but not with fire, while many studies reported a pH increase after fire (Muñoz‐Rojas et al., 2016; Ponder et al., 2009; Rodríguez et al., 2017; Yinghua et al., 2012). These studies attributed such a change in pH to ashes that release cations such as Ca^2+^ on the soil surface (Ponder et al., 2009; Rodríguez et al., 2017). It is not clear why this mechanism does not apply to Lamto savanna. The higher NH_4_^+^ concentration after the fire is likely due to the deposition of ashes (Xue et al., 2014).

The total C and N contents were higher under trees and grasses than in bare soil patches, as expected since bare soil patches have been deprived of plant litter and exudate inputs for 1 year before soil sampling. The NH_4_^+^ concentration was higher under trees than under grasses and bare soil. This has already been reported by Srikanthasamy et al. (2018) and Mordelet et al. (1993) and could be due to a general enrichment in organic matter and mineral nutrients under trees, which is a rather general pattern in savannas (Kellman, 1979; Ludwig et al., 2004) even in savannas where trees are not N‐fixing legumes (such as Lamto). The underlying mechanisms are not fully clear but several mechanisms have been proposed: (a) Fire intensity decreases below tree clump (Abbadie et al., 2006), which could lead to lower losses of organic matter and mineral nutrients, (b) tree roots can go deeper than grass roots inside soils and could absorb mineral nutrients (e.g., phosphorus) in deep soil layer and bring it to the soil surface (Schroth & Zech, 1995), and (c) tree roots extend beyond tree canopy, which allows them to forage mineral nutrients from a large area and concentrate them below the canopy (Ludwig et al., 2004).

The total archaeal and bacterial abundances did not change with the vegetation cover and the fire, but the total fungal abundance was higher under trees and grasses than in bare soil and was not impacted by fire. This suggests either a general decrease in wood‐decay and litter‐decomposing fungi linked to lower organic matter in bare soil patches, and/or a strong rhizospheric effect due to associations between mycorrhizal fungi and tree/grass roots (Vieira Junior et al., 2020; Vieira et al., 2020). However, no data are so far available on the abundance of mycorrhizae in Lamto savanna to confirm this hypothesis. Furthermore, the soil water content is lower under bare soil after the fire and soil water content is anyway low during the dry season. This could negatively impact the fungal abundance because (a) the impact of fire increases when the soil water content is lower (Beyers et al., 2005) and (b) drought is an important limiting factor for fungal growth in burned soils (Rutigliano et al., 2007). Bacterial and fungal transcript abundances increased after the fire, whatever the vegetation cover. This is probably due to the increase in mineral N availability after the fire. This can also be due to the fact that grasses (Gignoux et al., 2006 and personal observation) and trees resume their growth and probably root exudation after fire (Ricardo & Pons, 2001; Tessler et al., 2016; Wittenberg et al., 2007). Furthermore, disturbances tend to induce strong effects such as a decrease in hyphal length or triggered sporulation (Mataix‐Solera et al., 2002). As fungi are less heat‐tolerant than bacteria, the induced thermal stress could have stimulated activities linked to the response to disturbance, leading to an increase of the fungal transcript abundance. This hypothesis is supported by results of Srikanthasamy (2018): just after the fire, we have two times more fungal transcripts than during the rainy season (5 months after the fire).

As assumed, the total bacterial nitrifier abundance was lower under grasses due to the inhibition of nitrification (Lata et al., 2000; Srikanthasamy et al., 2018). Bacterial nitrifier transcripts were not detected before and after fire confirming that bacterial nitrifiers are not the main actors in the nitrification process in this savanna (Srikanthasamy, 2018). As previously reported (Srikanthasamy et al., 2018), the archaeal nitrifiers had a very low abundance under grasses in accordance with the inhibition of nitrification exerted by these grasses. Archaeal nitrifier transcripts were detected in all soil samples while bacterial nitrifier transcripts were detected in only one sample, so that we can suppose that archaeal nitrifiers are mainly responsible for nitrification in this savanna. The decrease in archaeal nitrifier transcripts after fire can be explained by two complementary mechanisms: (a) NH_4_^+^ concentration in the soil increases after the fire. Indeed, in the literature, archaeal nitrifier predominance and activities are principally controlled by two parameters: pH (that is not influenced by fire in the present study) and ammonium availability (Prosser & Nicol, 2012). Archaeal nitrifiers are less active with high pH and high ammonium concentrations (Cao et al., 2011; Nicol et al., 2008; Prosser & Nicol, 2012). (b) The intensity of nitrification inhibition by grasses likely increases after fire (NEA decreases to zero). Indeed, grasses start to grow quickly after fire so that their root exudation and their BNI activities probably also increase after the fire. Taken together, the nitrification enzyme activities and the archaeal nitrifiers transcriptional activities both decrease after fire. This further supports the hypothesis that archaeal nitrifiers are more influential for nitrification than bacterial nitrifiers in this savanna (Srikanthasamy, 2018).

Contrary to our hypothesis, very few interactions between the vegetation cover and the short‐term effect of fire significantly impacted the measured variables. This is particular the case for NEA, and the archaeal and bacterial nitrifiers abundances. This is probably due to the fact that, in our study, the impacts of the vegetation cover are much stronger than the short‐term impacts of fire, so that even when there is a significant fire effect (e.g., for NEA), this effect does not modify the effect of the vegetation. Indeed trees, grasses, and bare soil patches had contrasting effects on nitrification after fire (decrease for bare soil, drop to zero under grasses, stable under trees—Table S2).

## 5 CONCLUSIONS

Fires are known to be critical for the maintenance of savanna ecosystems (Bond et al., 2005). In particular, the impact of fires on tree–grass coexistence has thoroughly been studied (Bond et al., 2005; N'Dri, 2012; Sankaran et al., 2008). The impacts of fires on both soil properties and soil microorganisms are also widely acknowledged but have not been fully documented especially in African savannas (but see Belmok et al., 2019; Ferreira De Araújo et al., 2017). In the long‐term, the cumulative short‐term impacts of fires on mineral nutrient, C cycling and plant growth alter soil organic C concentration, pH, mineral nutrient concentrations, texture and structure, and water infiltration (Francos et al., 2018). It can also alter soil microbial activities and functional diversity (Yinghua et al., 2012). This is particularly true in the top 15 cm of soil where most aerobic microbial activities occur (Docherty et al., 2012) and that can be greatly impacted in cases of severe fires (Bárcenas‐Moreno et al., 2011). The short‐term impacts of fires and underlying mechanisms could be further studied by mesocosm experiments manipulating independently the involved factors (increase in temperature at the soil surface, addition of ashes, inputs of root exudates…). Studying both the short‐term (with these mesocosm experiments and field experiments) and the long‐term impacts of fires would benefit from documenting more precisely microbial diversity (through sequencing methods) because total abundances, total diversity, and the abundances of all taxa are likely to respond differently to fires. In the case of this savanna, the fire has an impact on the nitrifier communities, and Belmok et al. (2019) also reported that fires increased in the long term the diversity of archaeal *amoA* in the Brazilian Cerrado. An overall study of savanna present diversity, abundance and expression of nitrifier communities, will allow a better understanding of the impact of fire on nitrogen cycle.

## CONFLICT OF INTEREST

The authors declare that they have no conflicts of interest.

## AUTHOR CONTRIBUTIONS

**Tharaniya Srikanthasamy:** Conceptualization (lead); data curation (lead); formal analysis (equal); funding acquisition (supporting); investigation (equal); methodology (lead); project administration (supporting); resources (equal); software (equal); supervision (equal); validation (lead); visualization (equal); writing‐original draft (lead); writing‐review & editing (equal). **Sébastien Barot:** Conceptualization (equal); data curation (equal); formal analysis (equal); funding acquisition (supporting); investigation (equal); methodology (supporting); project administration (equal); resources (equal); software (equal); supervision (equal); validation (equal); visualization (equal); writing‐original draft (equal); writing‐review & editing (equal). **Fulgence K. Koffi:** Resources (supporting); writing‐original draft (supporting). **Kevin Tambosco:** Methodology (supporting). **Yoan Marcangeli:** Methodology (supporting). **David Carmignac:** Methodology (supporting). **Aya Brigitte N'Dri:** Methodology (equal); resources (supporting). **Jonathan Gervaix:** Methodology (supporting). **Xavier Le Roux:** Writing‐original draft (supporting). **Jean‐Christophe Lata:** Conceptualization (equal); data curation (equal); formal analysis (equal); funding acquisition (lead); investigation (equal); methodology (equal); project administration (lead); resources (equal); software (supporting); supervision (lead); validation (equal); visualization (equal); writing‐original draft (equal).

## Supporting information

Supplementary Material

## Data Availability

Data available on dryad: https://datadryad.org/stash/share/jnja6aVDlEH_mU8GYI4‐Km‐VpdchwN2y6mvmGGyJla4.
