# Supplementary material for: Short‐term impact of fire on the total soil microbial and nitrifier communities in a wet savanna
Source: Ecol Evol. 2021 Jul 1;11(15):9958–69. doi: 10.1002/ece3.7661 (PMC8328428; doi:10.1002/ece3.7661)
Supplement: Supplementary file 1 — Supplementary Material [file ECE3-11-9958-s001.docx]

**TABLE S1**: Real-Time PCR conditions used in our study

|  | Starting DNA concentration (ng.µL^-1^) | Starting RNA concentration (ng.µL^-1^) | Amplification efficiency (%) | qPCR conditions | Primer set | Reference |
| --- | --- | --- | --- | --- | --- | --- |
| Total archaea (16S rRNA) | 0.2 and 0.02 | 0.2 and 0.02 | 91.7 | 94°C-4min/(94°C-30 s/57°C–40s/72°C-40s)*40cycles | Parch519F  Arc915R | (Herfort et al., 2007) |
| Total bacteria (16S rRNA) | 2.0 and 0.2 | 0.2 and 0.02 | 95.2 | 95°C-3min/(95°C-15s/60°C–30s/72°C-30s)*40cycles | BAC341F  BAC515R | (Bru et al., 2011) |
| Total fungi (18S rRNA) | 2.0 and 0.2 | 2.0 and 0.2 | 85.2 | 95°C-10min/(95°C-15s/55°C–30s/72°C-30s)*40cycles | FR1  FR390 | (Vainio and Hantula, 2000) |
| Crenarchaeotal *amoA-AOA* gene | 20 | 8.0 | 84.1 | 95°C-3min/(95°C-15s/56°C–30s/72°C-30s)*35cycles | crenamoA23F - crenamoA616R | (Tourna et al., 2008) |
| Bacterial *amoA-AOB* gene | 20 | 8.0 | 91.8 | 95°C-3min/(95°C-15s/55°C–30s/72°C-30s)*40cycles | AmoA1F  AmoA2R | (Rotthauwe et al., 1997) |

Bru, D., Ramette, A., Saby, N.P., Dequiedt, S., Ranjard, L., Jolivet, C., Arrouays, D., Philippot, L., 2011. Determinants of the distribution of nitrogen-cycling microbial communities at the landscape scale. Isme Journal 5, 532–542. doi:10.1038/ismej.2010.130

Herfort, L., Schouten, S., Abbas, B., Veldhuis, M.J.W., Coolen, M.J.L., Wuchter, C., 2007. Variations in spatial and temporal distribution of Archaea in the North Sea in relation to environmental variables 62, 242–257. doi:10.1111/j.1574-6941.2007.00397.x

Rotthauwe, J.H., Witzel, K.P., Liesack, W., 1997. The ammonia monooxygenase structural gene amoa as a functional marker: Molecular fine-scale analysis of natural ammonia-oxidizing populations. Applied and Environmental Microbiology 63, 4704–4712. doi:10.1128/AEM.NA

Tourna, M., Freitag, T.E., Nicol, G.W., Prosser, J.I., 2008. Growth, activity and temperature responses of ammonia-oxidizing archaea and bacteria in soil microcosms. Environmental Microbiology 10, 1357–1364. doi:10.1111/j.1462-2920.2007.01563.x

Vainio, E.J., Hantula, J., 2000. Direct analysis of wood-inhabiting fungi using denaturing gradient gel electrophoresis of amplified ribosomal DNA. Mycological Research 104, 927–936. doi:10.1017/S0953756200002471

**TABLE S2** Soil physicochemical characteristics before (BF) and after (AF) the fire under different vegetation cover (BS = bare soil, GRA = Grass and TRE = Tree). NEA = nitrifying enzyme activities. Means±SE were calculated on five replicates

| Fire | Vegetation cover | NEA (µg N-(NO_3_^-^ + NO_2_^-^) h^-1^ g^-1^ dry soil) | [N-NO_3_^-^] (mg g^-1^ dry soil) | [N-NH_4_^+^] (mg g^-1^ dry soil) | Water content (%) | pH | Total N (%) | Total C (%) |
| --- | --- | --- | --- | --- | --- | --- | --- | --- |
| BF | BS | 14.62 ±1.27×10^-3^ | 12.66±0.24×10^-3^ | 5.55±0. 14×10^-3^ | 15.66±1.10 | 6.69±0.03 | 0.056±0.002 | 0.835±0.028 |
| AF | BS | 4.40±1.34×10^-3^ | 12.23±0.20×10^-3^ | 5.22±0.16×10^-3^ | 7.00±0.63 | 6.69±0.05 | 0.049±0.007 | 0.771±0.095 |
| BF | GRA | 1.92±1.07×10^-3^ | 12.27±0.16×10^-3^ | 6.12±0.27×10^-3^ | 12.18±1.23 | 6.64±0.06 | 0.065±0.003 | 1.057±0.070 |
| AF | GRA | NA | 12.89±0.13×10^-3^ | 7.15±0.28×10^-3^ | 10.94±1.19 | 6.66±0.04 | 0.057±0.005 | 0.941± 0.073 |
| BF | TRE | 21.89 ±5.20×10^-3^ | 12.60±0.19×10^-3^ | 6.41±0.18×10^-3^ | 16.56±0.59 | 6.87±0.03 | 0.072±0.003 | 0.992±0.028 |
| AF | TRE | 20.40±3.83×10^-3^ | 12.41±0.22×10^-3^ | 7.79±0.30×10^-3^ | 10.70±1.81 | 6.89±0.04 | 0.078±0.005 | 1.130±0.064 |

**TABLE S3** Abundances of DNA (archaeal 16S rRNA, bacterial 16S rRNA, fungal 18S rRNA, amoA-AOA, amoA-AOB) together with the relative abundances of archaea relatively to the whole abundance of bacteria and archaea, the ratio of nitrifying archaea (bacteria) over all archaea (bacteria) and the bacteria/fungi ratio. Means and standard errors were calculated from five replicates for all combinations of fire impact (before (BF) and after (AF)) and vegetation cover (BS = bare soil, GRA = Grass and TRE = Tree)

| Fire | Vegetation cover | Total archaea (16S rRNA)  (copies g^-1^ dry soil) | Total bacteria (16S rRNA) (copies g^-1^ dry soil) | Total fungi (18S rRNA) (copies g^-1^ dry soil) | Bacteria/Fungi ratio | AOA  (copies g^-1^ dry soil) | AOB  (copies g^-1^ dry soil) | AOA/(AOA+AOB) ratio | AOA/Archaea ratio | AOB/Bacteria ratio |
| --- | --- | --- | --- | --- | --- | --- | --- | --- | --- | --- |
| BF | BS | 3.00±0.30×10^9^ | 1.81±0.20×10^9^ | 1.91±0.24×10^8^ | 9.86±1.15 | 1.26±0.36×10^6^ | 6.31±2.82×10^5^ | 0.67±0.07 | 3.86±0.92×10^-4^ | 3.61±1.69×10^-4^ |
| AF | BS | 2.52±0.10×10^9^ | 1.70±0.09×10^9^ | 1.70±0.05×10^8^ | 9.97±0.42 | 9.87±2.37×10^5^ | 1.23±0.42×10^6^ | 0.47±0.12 | 3.81±0.85×10^-4^ | 7.29±2.50×10^-4^ |
| BF | GRA | 3.60±0.27×10^9^ | 1.71±0.19×10^9^ | 7.47±1.50×10^8^ | 2.60±0.51 | NA | 1.62±0.43×10^5^ | NA | NA | 1.07±0.07×10^-4^ |
| BF | GRA | 2,38±1.25×10^9^ | 1.71±0.19×10^9^ | 7.47±1.50×10^8^ | 2.60±0.51 | NA | 1.62±0.43×10^5^ | NA | NA | 1.07±0.07×10^-4^ |
| AF | GRA | 3.60±0.27×10^9^ | 1.46±0.23×10^9^ | 8.98±1.92×10^8^ | 1.97±0.51 | NA | 1.33±0.43×10^5^ | NA | NA | 8.59±0.16×10^-4^ |
| BF | TRE | 2.40±0.52×10^9^ | 1.76±0.41×10^9^ | 7.19±0.16×10^8^ | 2.55±0.31 | 1.10±0.37×10^6^ | 3.44±0.37×10^5^ | 0.69±0.08 | 4.63±0.99×10^-4^ | 3.02±1.28×10^-4^ |
| AF | TRE | 2.94±0.52×10^9^ | 2.19±0.45×10^9^ | 8.05±0.11×10^8^ | 3.06±0.78 | 1.80±0.56×10^6^ | 8.14±2.64×10^5^ | 0.69±0.03 | 7.50±1.49×10^-4^ | 4.35±0.73×10^-4^ |

**TABLE S4** Abundances of transcripts (archaeal 16S rRNA, bacterial 16S rRNA, fungal 18S rRNA, amoA-AOA, amoA-AOB) and ratios of amoA-AOA (amoA-AOB) transcript abundance over the whole abundance of archaeal (bacterial) transcripts. Means and standard errors were calculated on five replicates for all combinations of fire impact (before (BF) and after (AF)) and vegetation cover (BS = bare soil, GRA = Grass and TRE = Tree)

| Fire | Vegetation cover | Total archaea transcripts (16S rRNA)  (copies g^-1^ dry soil) | Total bacteria transcripts (16S rRNA) (copies g^-1^ dry soil) | Total fungi transcripts (18S rRNA) (copies g^-1^ dry soil) | Bacteria/Fungi transcripts ratio | AOA transcripts  (copies g^-1^ dry soil) | AOB transcripts  (copies g^-1^ dry soil) | AOA/Archaea transcripts ratio |
| --- | --- | --- | --- | --- | --- | --- | --- | --- |
| BF | BS | 1.05±0.19× 10^10^ | 2.97±0.37 × 10^9^ | 1.79±0.81× 10^7^ | 2.26±1.45× 10^3^ | 3.34±1.04× 10^4^ | NA | 3.79±1.34× 10^-6^ |
| AF | BS | 0.36±0.10× 10^10^ | 7.14±2.44 × 10^9^ | 7.86±3.39× 10^8^ | 1.14±0.32× 10^1^ | NA | NA | NA |
| BF | GRA | 1.10±0.40× 10^10^ | 3.78±0.96× 10^9^ | 0.925±0.83× 10^8^ | 1.70±0.80× 10^2^ | 1.68±0.91× 10^5^ | NA | 0.53±0.53× 10^1^ |
| AF | GRA | 1.73±0.44× 10^10^ | 3.10±0.72× 10^10^ | 7.68±2.09× 10^9^ | 4.13±0.37×10^0^ | 4.91±3.28× 10^3^ | NA | 2.58±1.67× 10^-6^ |
| BF | TRE | 1.42±0.22× 10^10^ | 3.58±0.85× 10^9^ | 3.68±2.40× 10^8^ | 2.08±1.55× 10^2^ | 4.21±1.01× 10^4^ | NA | 3.26±0.99× 10^-6^ |
| AF | TRE | 1.09±0.25× 10^10^ | 1.71±0.47× 10^10^ | 4.71±1.42× 10^9^ | 3.76±0.16× 10^0^ | 1.72±0.40× 10^4^ | NA | 2.14±0.71× 10^-6^ |

**FIGURE S1** Correlation circle of the principal component analysis (PCA) on soil properties. NEA= nitrifying enzyme activity; SWC=soil water content; Ctot and Ntot=total C and N soil content; NO3 and NH4=soil nitrate and ammonium concentrations

**FIGURE S2** Biplot of the principal component analysis (PCA) on soil properties comparing the different vegetation covers: bare soil (BS, in yellow), dominant grass species *Hyparrhenia diplandra* (GRA, in green) and dominant tree species *Crossopteryx febrifuga* (TRE, brown). NEA= nitrifying enzyme activity; SWC=soil water content; Ctot and Ntot=total C and N soil content; NO3 and NH4=soil nitrate and ammonium concentrations

**FIGURE S3** Biplot of the principal component analysis on soil properties comparing the two fire treatments: before fire (BF, in yellow) and after fire (AF, in red). NEA= nitrifying enzyme activity; SWC=soil water content; Ctot and Ntot=total C and N soil content; NO3 and NH4=soil nitrate and ammonium concentrations
